# Dr. A. Crandall Way

**Published:** 1916-12

**Authors:** 


### OBITUARY

Readers are requested to report promptly the death of all physicians in Western New York, or former residents of this region, or graduates of any medical school in Western New York, and to notify the families of the deceased of our desire to publish adequate Obituary notices.

**Dr. A. Crandall Way,** Buffalo 1895, a prominent practitioner of Perry, N. Y., died of tuberculosis complicating diabetes of some years' standing, at Saranac Lake, Nov. 6. He was born at Binghamton, Aug. 28, 1870. He began his medical course at the University of Michigan but completed it at Buffalo and began practice in that city, later moving to Perry Center and more recently to Perry, as more convenient for his radius of practice. The funeral was held at the residence of his parents, Mr. and Mrs. D. A. Way who, with his wife, Mrs. Florence Morse Way and an infant daughter, Carroll, survive him. Interment was in the Binghamton cemetery, the services being in charge of the Elks' Lodge of that city. Dr. Way was also a Mason. He was not only active in practice but a regular attendant at state and national medical meetings. His pancreatic lesion interfered in no way with his duties and sunny disposition and his prudence prevented it from affecting his general health seriously till last winter when, following an attack of influenza, signs of pulmonary tuberculosis developed. After a southern trip, he went to Saranac Lake and made a valiant but vain fight to recover his health. He will be missed by a large circle of devoted patients and friends.

